# Chalfant’s Pardon

**Published:** 1886-03

**Authors:** 


### Chalfant's Pardon.

The following item of news appears in the *Dental Cosmos* for October. It is from the Sacramento *Bee*, of late date :

Governor Stoneman to-day issued a pardon to Dr. Samuel B. Chalfant, who killed Josiah Bacon, an agent of the Goodyear Rubber Company, in the Baldwin Hotel, San Francisco, several years ago, and who was convicted of murder in the second degree and sentenced to ten years' imprisonment at San Quentin. It will be rembered that shortly after Chalfant was imprisoned, a woman named Perkins appeared on the scene and began efforts to secure his release. At one time the doctor escaped from the prison, through plans believed to have been matured by the woman. He was recaptured in Nevada, however, and returned to San Quentin. Mrs. Perkins never abated her efforts to secure his pardon, and how successfully she has labored is shown by the fact that his pardon was petitioned for by the entire jury before whom Chalfant was tried, the judge who sentenced him, the prosecuting attorney, and a large number of the leading citizens of San Francisco. It is understood that Mrs. Perkins will soon be come Mrs. Chalfant.

A London paper says that an apothecary of Thorndale had just received a fresh supply of vaccine points, and some of them happened to be exposed to view on his counter. A burly farmer from that neighborhood was in at the time, and amused himself by using one of the points as a tooth-pick, pricking his gums in the operation. It "took" in the most approved style, and the man is now in possession of a mouth that is crowding all the other features of his face out of shape.

Chronic Ulcers.—Dr. W. P. Howe, of Charleston, Mo., writes to us saying, " that *lime* procured from barrels that have stood open for some time, sprinkled upon chronic ulcers will cure them when everything else, usually prescribed fails. * * * Cleanse the ulcer with warm water and soap once every two days and fill the sore with lime; no more bad odor, no more pain, and a remedy so cheap that it is within the reach of everyone.

